# Acute Effects of High-Speed Repetitive Loading on Achilles Tendon Viscoelasticity and Ankle Agonist–Antagonist Co-Contraction: A Combined MyotonPRO and Surface Electromyography Analysis in Male Collegiate American Football Players

**DOI:** 10.3390/jfmk11030361

**Published:** 2026-09-09

**Authors:** Shuho Kang, Ja Yeon Lee, Ji Hwan Jeong, Chae Kwan Lee, Ilbong Park

**Affiliations:** 1 Department of Sports Rehabilitation, Busan University of Foreign Studies, Busan 46234, Republic of Korea; 20236204@bufs.ac.kr (S.K.); 20236202@bufs.ac.kr (J.Y.L.); 20246001@bufs.ac.kr (C.K.L.); 2 Department of Convergence Sports Instruction, Hwashin Cyber University, Busan 47598, Republic of Korea; rehabpd@hscu.ac.kr

**Keywords:** Achilles tendon, viscoelasticity, decrement, myotonometry, MyotonPRO, surface electromyography, co-contraction, stretch–shortening cycle, American football

## Abstract

**Background:** Achilles tendon viscoelasticity and ankle neuromuscular co-contraction are functionally linked, yet how they jointly adapt during brief high-speed repetitive loading remains unclear. We simultaneously quantified Achilles tendon viscoelasticity (MyotonPRO) and ankle agonist–antagonist co-contraction (surface electromyography, sEMG) under two stance conditions and three repetition counts. **Methods:** Twenty-seven active male collegiate American football players (23.19 ± 2.63 years) performed rapid heel-raise/landing cycles in a bilateral ground stance (BGS) and a unilateral box stance (UBS) at 1, 5, and 10 repetitions. Tendon decrement (D), stiffness (S), and tone (F), the co-activation index (CoAct), and the co-contraction index (CCI) were analyzed using two-way (2 × 3) repeated-measures ANOVA. **Results:** D decreased significantly as repetitions increased (pooled across stance conditions: 1 rep 0.973 → 10 reps 0.937; F (2, 52) = 4.68, *p* = 0.014, ηp^2^ = 0.153), indicating improved elastic-recovery efficiency, whereas S and F remained stable. CoAct was maintained constantly at ≈72% regardless of stance or repetition (all *p* > 0.05), and the medial gastrocnemius was consistently activated first (agonist-led feedforward priming). Tissue-level elastic adaptation thus occurred without a concurrent change in neuromuscular co-contraction—a tissue–neural dissociation confirmed in both change scores and correlation structure. **Conclusions:** Acute enhancement of Achilles tendon elasticity during high-speed repetition appears to be a peripheral, tissue-level mechanical phenomenon expressed independently of neuromuscular co-contraction. Combined myotonometric–sEMG assessment reveals functional information not captured by imaging alone.

## 1. Introduction

The Achilles tendon is the strongest and thickest tendon in the human body. It transmits the contractile force of the triceps surae, comprising the gastrocnemius and soleus to the calcaneus and thereby serves as a central mechanism for propulsive actions such as walking, running, and jumping [1]. Rather than a passive connective structure, it functions as an active functional unit that stores and releases elastic energy across loading cycles and operates as a key determinant of the mechanical efficiency of muscle contraction within the muscle–tendon unit (MTU) [2,3]. In particular, during explosive, stretch–shortening cycle (SSC)-based movements, the viscoelasticity of the Achilles tendon is directly related to performance, and its mechanical properties are reorganized in a variable manner according to loading intensity, training history, and postural conditions [4,5].

However, this high functionality simultaneously entails susceptibility to injury. Achilles tendon lesions are among the most frequently reported overuse injuries in athletes whose sports involve repeated jumping, landing, and change-of-direction movements; when they become chronic, they are accompanied by disorganization of the collagen fiber arrangement, reduced viscoelasticity, and maladaptive changes in stiffness, cumulatively aggravating the risk of re-injury [6,7,8,9]. Therefore, quantitatively evaluating the mechanical adaptation of the Achilles tendon to changing load conditions during exercise has clinical and field value for both injury prevention and performance optimization.

Traditionally, the mechanical properties of the Achilles tendon have been measured using ultrasound imaging and shear-wave elastography. However, such imaging-based techniques require costly equipment and skilled operators and have intrinsic limitations in field applicability and the efficiency of repeated measurement [10]. As a complementary alternative, the portable myotonometric device MyotonPRO has attracted attention. By analyzing the damped oscillation of tissue in response to a brief mechanical impulse, it non-invasively derives multidimensional viscoelastic variables including stiffness (N/m), elasticity (logarithmic decrement of damping), and tone (frequency) [11]. In numerous prior studies, the MyotonPRO has demonstrated excellent intra- and inter-rater reliability [12,13,14], and its validity has been reinforced by recently reported significant correlations with ultrasound-based measures such as shear-wave elastography [10].

On this basis, the MyotonPRO has been used in diverse studies, including the assessment of Achilles tendon mechanical properties in static postures, the clinical discrimination of patients with tendinopathy, and the tracking of training effects [15].

Meanwhile, changes in the mechanical properties of the Achilles tendon do not occur independently but emerge in close coupling with the neuromuscular control strategies of the agonist and antagonist muscles surrounding the ankle joint [16,17]. In particular, the medial gastrocnemius (MG), the lateral gastrocnemius (LG), and the tibialis anterior (TA) form a representative antagonistic pair for ankle plantar–dorsiflexion, and the co-contraction of these two muscle groups functions as a core mechanism for joint stability and postural control [18]. Co-contraction provides stability while entailing energetic inefficiency. Whether, in relatively brief high-speed movements, co-contraction varies progressively with repetition count or is maintained at a constant level, and how such neuromuscular activation patterns are coupled with changes in Achilles tendon viscoelasticity, remain unresolved.

Moreover, even for the same task, individuals may differ in execution speed. Considering that in explosive SSC-based movements the storage and release of elastic energy occur primarily in the tendon [2,4], the present study additionally measured movement time and exploratorily examined differences in viscoelasticity and co-contraction according to execution speed.

Therefore, to accurately interpret the mechanical adaptation of the Achilles tendon under changing postural and loading conditions, it is necessary to simultaneously quantify the level of neuromuscular co-contraction expressed under identical conditions and to analyze the tissue level and the neuromuscular level in an integrated manner.

To address this gap, the present study aimed to simultaneously quantify, within the same participants, Achilles tendon viscoelasticity (stiffness, elasticity, tone) measured by MyotonPRO and the activation and co-contraction of ankle agonist–antagonist muscles measured by surface electromyography, by combining two stance conditions and three repetition counts (1, 5, and 10). Specifically, the study examined (1) changes in Achilles tendon viscoelasticity according to stance condition and repetition count, (2) the pattern of agonist–antagonist co-contraction, and (3) the relationship between tissue-level viscoelastic adaptation and neuromuscular-level co-contraction; and, exploratorily, (4) how movement time (execution speed) is coupled with Achilles tendon elasticity. We hypothesized that as high-speed repetitions accumulate, Achilles tendon elasticity would improve (i.e., the elastic decrement would decrease); we explored whether this tissue-level adaptation is accompanied by changes in co-contraction or is expressed upon a constant neuromuscular base; and we hypothesized that participants with shorter movement times (faster execution speed) would exhibit higher Achilles tendon elasticity. In addition, because the UBS condition relies on a single-limb support base and is therefore inherently less stable than the bilateral BGS condition, we hypothesized that UBS would elicit greater ankle agonist–antagonist co-contraction and a correspondingly larger change in Achilles tendon viscoelasticity than BGS, reflecting greater stability-driven neuromuscular and tissue-level demand.

The distinctive significance of this study lies in two aspects. First, instead of ultrasound-based morphological variables, it adopts the multidimensional viscoelastic indices derived from the MyotonPRO—particularly the damping-based elastic loss and tissue tone that are difficult to capture directly with ultrasound. These stand in a complementary rather than a mutually exclusive relationship with ultrasound imaging, providing information in a dimension that existing imaging techniques cannot capture. Second, by simultaneously applying the MyotonPRO and surface electromyography under identical conditions, it analyzes tissue-level mechanical adaptation and the neuromuscular-level control strategy in an integrated manner. Such a multilayered approach offers the possibility of illuminating, from a new dimension, the functional regulatory mechanisms of the Achilles tendon that prior research has addressed only fragmentarily.

Ultimately, by providing empirical evidence on how changes in loading conditions differentiate the viscoelastic response of the Achilles tendon, this study may serve as foundational data for stance design and load-modulation strategies in sports settings and, further, for the development of Achilles tendon injury-prevention programs.

## 2. Materials and Methods

### 2.1. Participants

An a priori power analysis (G*Power 3.1.9.7) based on a repeated-measures ANOVA design (2 conditions × 3 repetition counts), with a medium effect size (f = 0.25), α = 0.05, and power of 0.80, indicated that a minimum of 24 participants was required. Allowing for potential dropout, 32 participants were recruited; 2 withdrew owing to scheduling failures, leaving 30 who participated in measurement. Of these, 3 in whom simultaneous surface electromyography measurement was not completed were excluded from the analysis, yielding a final analytic sample of 27 active male collegiate American football players. The final sample size (*n* = 27) exceeds the minimum (*n* = 24) derived from the power analysis.

Inclusion criteria were: (1) adult males aged ≥19 years who were active regular collegiate American football players; (2) participation in regular training ≥3 times per week; and (3) the ability to provide voluntary written consent to the study procedures. Exclusion criteria were: (1) a history of Achilles tendon, lower-leg, or ankle joint injury within the previous 6 months; (2) a diagnosis of chronic Achilles tendinopathy; (3) the use of medications that could affect measurement; (4) skin damage at the MyotonPRO or electromyography measurement sites; and (5) the inability to complete measurement owing to physical or psychological anxiety.

All participants were fully informed of the purpose, procedures, and potential risks of the study and submitted written informed consent. The study was approved by the Institutional Review Board and conducted in accordance with the principles of the Declaration of Helsinki (IRB approval no.: P01-202607-01-050; CRIS: KCT0012285). The general characteristics of the participants are presented in Table 1.

### 2.2. Experimental Design and Stance Conditions

**Figure 1:**
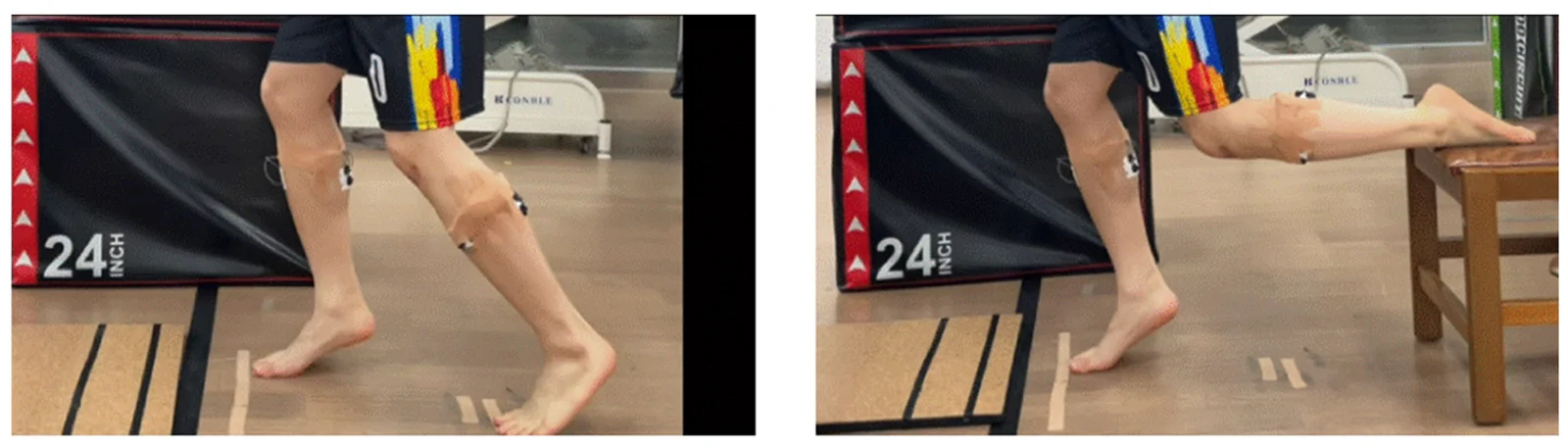
Experimental stance conditions: bilateral ground stance (BGS, (**left**)) and unilateral box stance (UBS, (**right**)). The dominant (front) foot performs the rapid heel-raise/landing cycle.

This study applied a within-subject repeated-measures design. On a single measurement day, each participant performed all combinations of two stance conditions and three repetition-count conditions, and all measurements were performed only on the dominant foot. The postural conditions during movement comprised two conditions, both based on a split stance but differing in the support-surface height of the rear lower limb, so that the loading condition imposed on the front dominant foot was differentiated (Figure 1).

(1) Bilateral ground stance (BGS). Participants adopted a split stance with the legs spread anteroposteriorly at a stride equal to their own tibial length, positioning the dominant foot anteriorly so that most of the body weight was borne on the front foot. The knee of the rear lower limb was lightly flexed to ≈20° to prevent excessive weight distribution to the rear foot. Using the anterior weight shift that naturally arises from loading the front foot, participants raised the heels of both feet to maintain the ankle joint in a neutral position.
(2) Unilateral box stance (UBS). Participants placed the non-dominant foot on a stable box 20 cm high to support the rear lower limb and positioned the dominant foot on the ground anterior to the box, adopting a split stance. With the rear lower limb on the box, the rear support point was 20 cm higher than the front support point; consequently, body weight was shifted maximally to the anterior dominant foot, increasing the proportion of load on the anterior single foot relative to the BGS condition. As in BGS, the heel of the dominant foot was raised, and the ankle was maintained in a neutral position.

**Figure 2:**
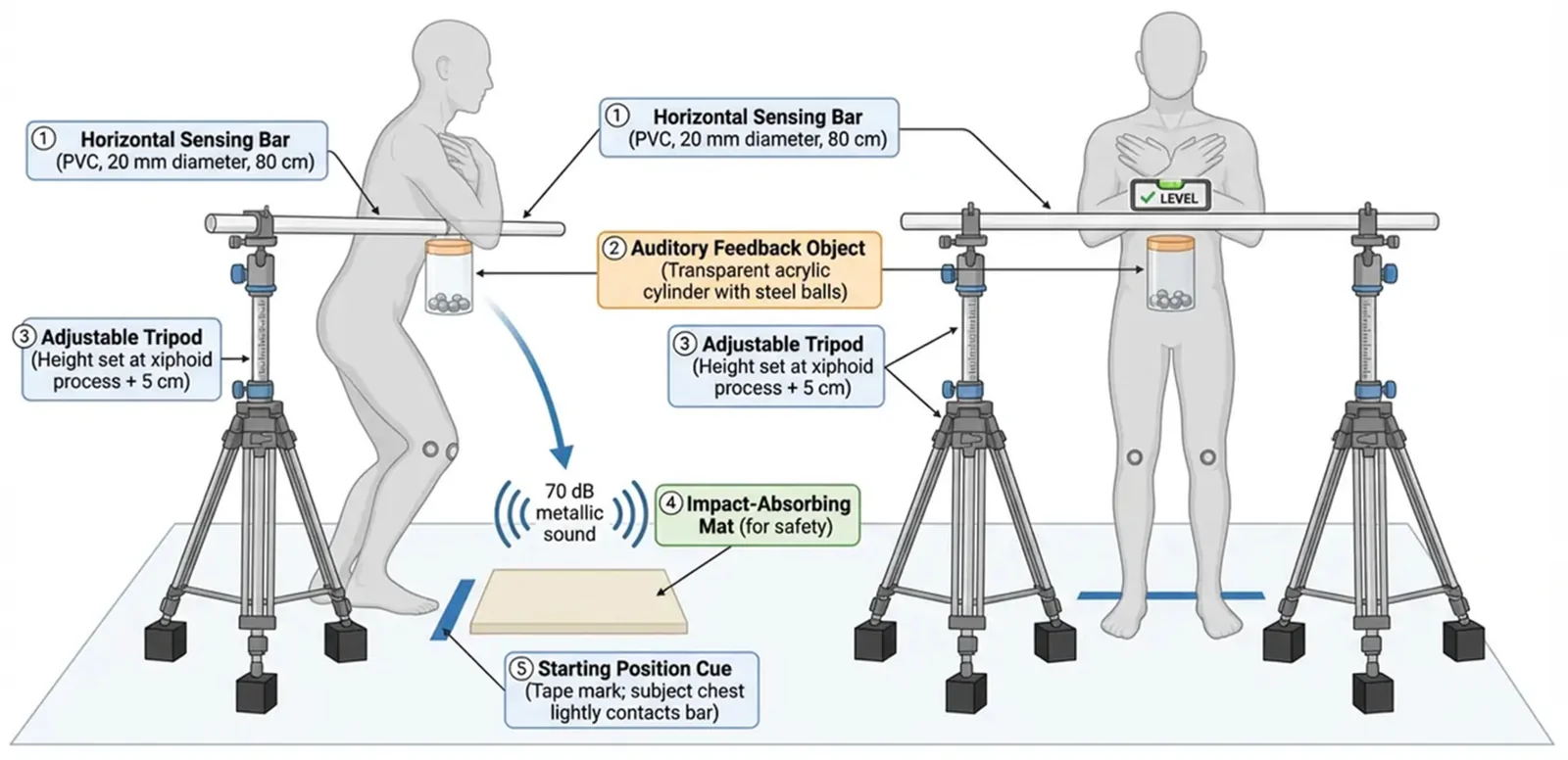
Height-adjustable auditory feedback rig for anterior–posterior trunk movement control. An acrylic container with steel balls provides immediate auditory feedback (~70 dB) upon contact with the horizontal sensing bar. This schematic was created by the authors using Microsoft PowerPoint (version 2408; Microsoft Corp., Redmond, WA, USA).

In both conditions, to minimize compensatory upper-limb movement, participants crossed and fixed both arms in front of the chest. To control anteroposterior deviation of the center of mass in real time, an auditory feedback device was installed (Figure 2): lightweight plastic bars of identical form were positioned 10 cm anterior to the chest and 10 cm posterior to the pelvis, respectively, and a bell was attached to each bar so that an auditory signal (~70 dB) was generated if a bar was pushed beyond a set threshold anteriorly or posteriorly.

Upon the start signal, participants rapidly raised the front foot and landed on a 2-cm-high landing platform located 5 cm anteriorly, then immediately returned to the starting posture; this was defined as one cycle. Movement was performed at each participant’s maximal achievable speed. In each stance condition, three repetition-count conditions—1, 5, and 10—were performed, and for safety reasons, BGS was performed first, followed by UBS. Within each stance, the three repetition-count conditions were always administered in this fixed order (1, then 5, then 10 repetitions) for every participant rather than being randomized (Section 4.7); each repetition-count condition was performed once, and the corresponding MyotonPRO reading reflects the device’s automatic mean of three consecutive mechanical impulses taken at that single time point (Section 2.3), not three repetitions of the behavioral task itself. To control landing accuracy, a 10-cm-wide target landing zone was marked inside the edge of a 50 cm × 50 cm platform, and participants were required to land accurately within this zone on every trial. A trial was treated as invalid and re-measured after sufficient rest if (i) the front foot completely missed the platform, (ii) landing occurred outside the target landing zone, or (iii) a control bar was pushed beyond threshold so that the bell rang (regarded as center-of-mass deviation). In total, three such re-measurements were required, all within the BGS condition and none in UBS: two arose from intermittent electromyography sensor contact during the 5-repetition condition and one during the 10-repetition condition; each was re-measured only after sufficient additional rest.

**Figure 3:**
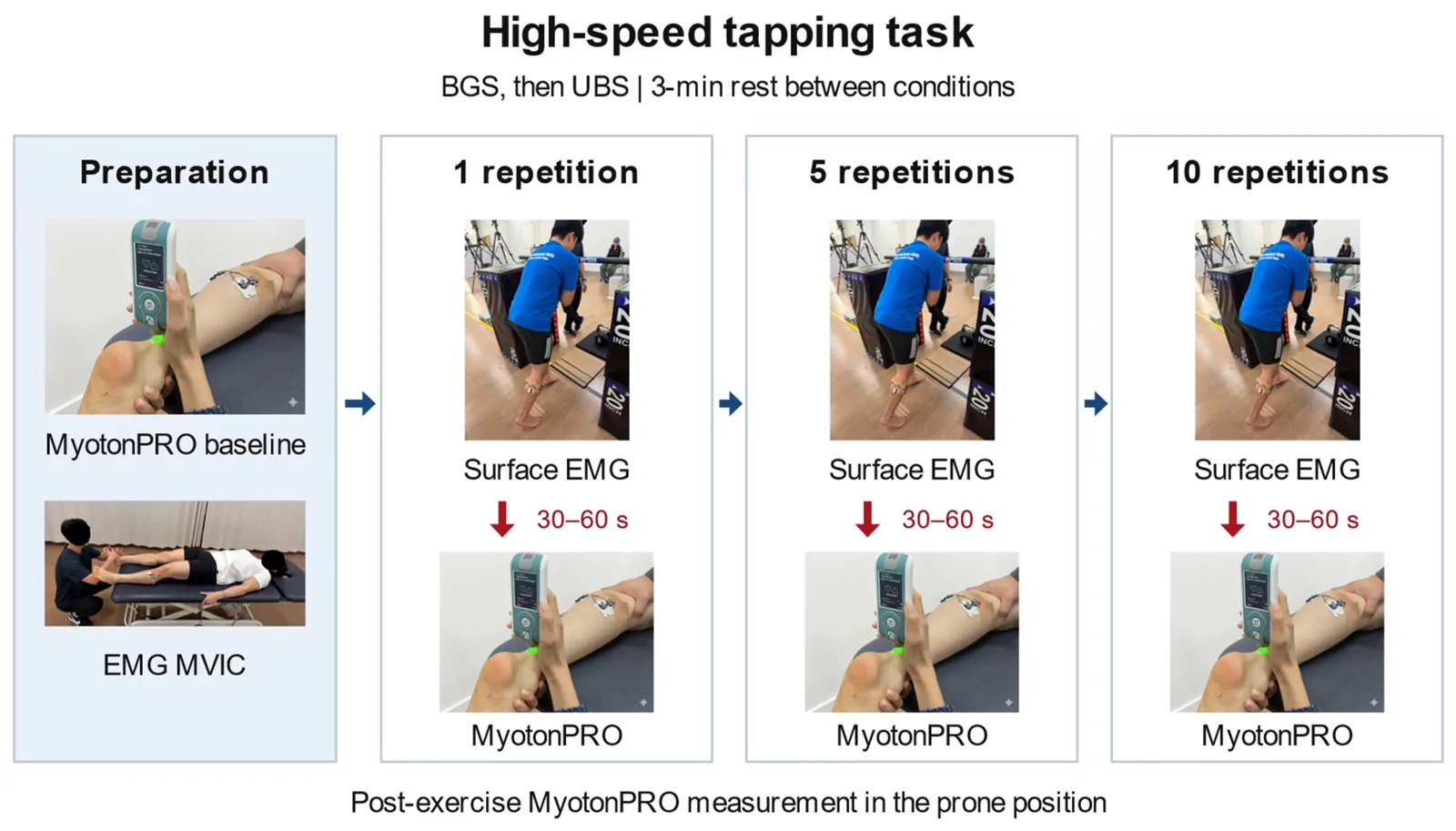
Overview of the high-speed tapping-task measurement protocol. After preparation (MyotonPRO baseline and EMG MVIC), participants performed the split-stance tapping task at 1, 5, and 10 repetitions; MyotonPRO and surface-EMG measurements were obtained at each step, with prone post-exercise measurement completed within 30–60 s and a 3-min rest between conditions.

MyotonPRO measurement was performed in the prone position at all time points. Immediately before (baseline) and immediately after each exercise condition, participants lay prone on the measurement bed with the ankle joint allowed to hang naturally beyond the edge of the bed, in a standardized posture. This posture allows the calf MTU to stabilize in a resting state and permits measurement of pure tissue viscoelasticity without stiffness fluctuations due to active muscle contraction [14,15]. The procedure was repeated for the six exercise conditions (BGS 1/5/10, UBS 1/5/10), yielding measurements at seven time points (1 baseline + 6 post-exercise). The transition time from the end of exercise to the start of prone measurement was kept within 30–60 s in all conditions, and the inter-condition rest was 3 min, consistent with the recovery between resistance-exercise sets recommended by the American College of Sports Medicine (2–5 min). The overall measurement protocol is summarized in Figure 3.

### 2.3. Measurements

**Figure 4:**
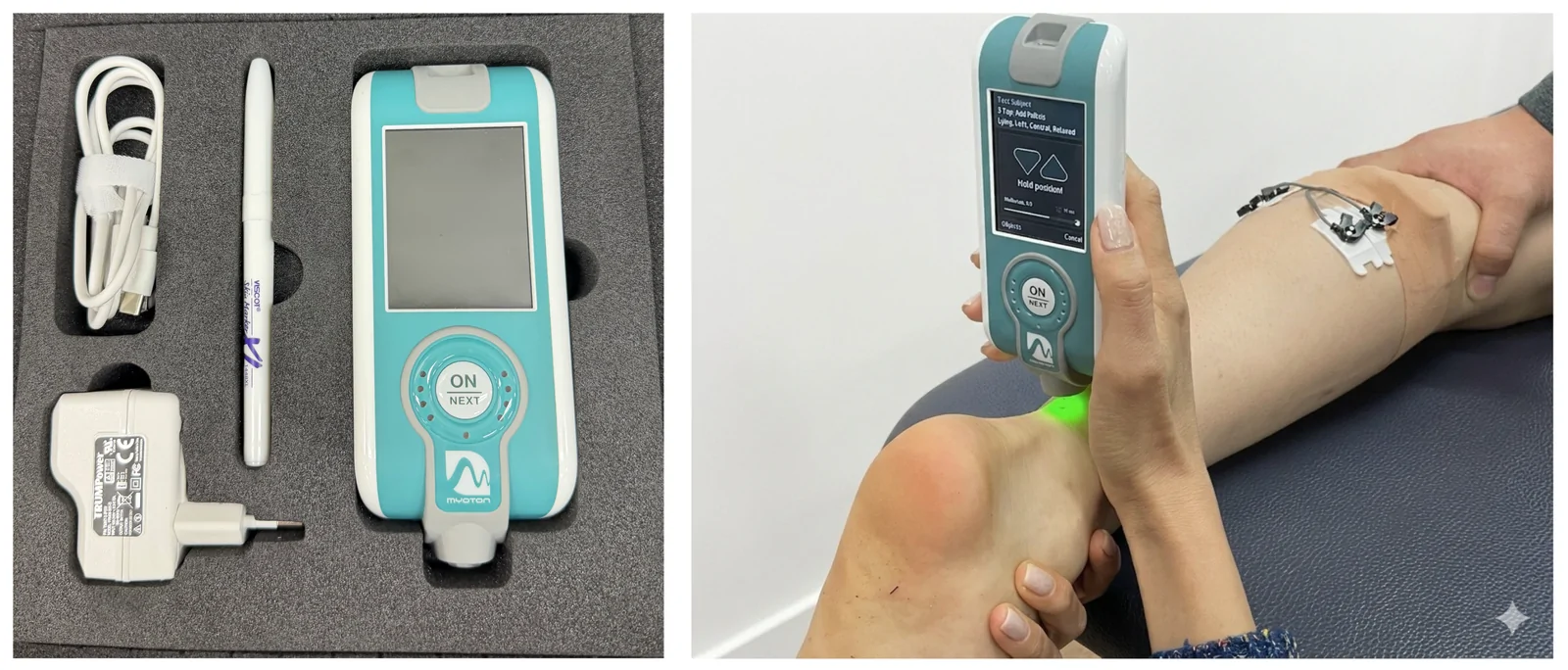
The portable MyotonPRO myotonometer (**left**) and measurement of the Achilles tendon in the prone position, with surface-EMG electrodes attached to the calf (**right**).

MyotonPRO Achilles tendon viscoelasticity. The viscoelastic properties of the Achilles tendon were measured using the portable myotonometric device MyotonPRO (Myoton AS, Tallinn, Estonia) (Figure 4). The device applies a brief mechanical impulse of ≈15 ms to the tissue, records the resulting damped natural oscillation with an accelerometer, and from the signal form derives multidimensional viscoelastic variables [11,12]: tone (F, Hz), the natural oscillation frequency reflecting the intrinsic resting tension of the tissue; stiffness (S, N/m), the tissue’s resistance to external deformation; and decrement (D, dimensionless), the logarithmic decrement of the damped oscillation, which directly represents the dissipation of mechanical energy during recovery after deformation, with a lower D indicating higher elastic-recovery efficiency. These three core variables (F, S, D) were adopted as the primary analysis variables.

The measurement site was the mid-portion of the dominant-foot Achilles tendon, 5 cm proximal to the calcaneal insertion; this point was marked and repeatedly measured at the same location (Figure 4, right). The probe was applied perpendicular to the skin by a single pre-trained examiner. Using the device’s automatic multi-impulse mode, three consecutive impulses were applied per measurement, and the mean was computed automatically. The tap time was adjusted to the recommended value by the device’s automatic detection function; according to the manufacturer, this is a normal correction mechanism ensuring that the time to reach maximum deformation is not exceeded, and it guarantees the validity of the measurement. Only data with a coefficient of variation (CV%) within 3% were accepted as valid. No formal minimal detectable change (MDC) value for the decrement (D) at the Achilles tendon has been established in the literature; although prior MyotonPRO reliability studies report generally high intra- and inter-rater reliability for its viscoelastic parameters [12,13,14], the modest magnitude of the group-level D change observed here (≈0.97 to ≈0.92) cannot be directly benchmarked against a validated measurement-error threshold, which is acknowledged as a limitation (Section 4.7).

Surface electromyography. Neuromuscular activation around the ankle was measured using a wireless surface electromyography (sEMG) system (Ultium EMG, Noraxon USA, Inc., Scottsdale, AZ, USA) on three muscles forming the principal antagonistic pair for ankle plantar–dorsiflexion: the medial gastrocnemius (MG), the lateral gastrocnemius (LG), and the tibialis anterior (TA). Electrode placement followed the SENIAM guidelines [19], with bipolar Ag/AgCl electrodes at a 20-mm inter-electrode distance. sEMG signals were sampled at 1000 Hz, band-pass filtered at 20–450 Hz, and smoothed with a 50-ms moving-window RMS algorithm. Activation was normalized to the maximum voluntary isometric contraction (%MVIC), applying manual resistance to the MG and LG in plantarflexion and to the TA in dorsiflexion from the ankle-neutral posture [20,21]; the higher RMS of two 3-s trials was used as the reference.

Before quantifying agonist–antagonist co-activation, the MG and LG activations were averaged into a single gastrocnemius (agonist) variable, as the two heads did not differ significantly (paired t-test, t(26) = 0.20, *p* = 0.840). The co-activation index (CoAct) was based on the method described in [16], computed in an amplitude-based form applicable to condition-wise mean normalized activation:

CoAct (%) = 2 × min(EMG_agonist, EMG_antagonist)/(EMG_agonist + EMG_antagonist) × 100.

Both CoAct and CCI were computed separately for each participant from their condition-wise mean normalized activation, and all reported values represent the mean of these participant-level indices rather than indices derived from group-averaged EMG. This index approaches 100% as the agonist and antagonist are activated together in a balanced manner. To complementarily quantify the magnitude of co-contraction, the co-contraction index (CCI) [22] was also computed, designating the lower- and higher-activation muscle among the agonist (gastrocnemius) and antagonist (tibialis anterior) as EMG_low and EMG_high:

CCI = (EMG_low/EMG_high) × (EMG_low + EMG_high).

Whereas CoAct reflects only the balance (0–100%) of the two muscles, CCI reflects both balance and total activation.

Movement time. Movement videos were recorded with a high-speed camera (NiNOX 120, Noraxon USA, Inc., Scottsdale, AZ, USA; 640 × 480, 120 fps), hardware-synchronized with the sEMG system so that video and EMG signals shared the same timeline in Noraxon MR4 software 3.20. Movement time (s) was derived by frame-by-frame marking of the start (the forefoot of the dominant foot leaving the ground) and end (the forefoot re-contacting the ground after landing and returning) points; the temporal resolution was ≈8.3 ms. Per-cycle movement time was the total time divided by the repetition count, based on the single trial performed for that repetition-count condition (Section 2.2). Because movement was instructed at maximal speed, a shorter movement time indicates faster (superior) performance.

### 2.4. Data Analysis

All analyses were performed in SPSS 26.0 (SPSS Inc., Chicago, IL, USA). Descriptive statistics are mean ± standard deviation (M ± SD). Normality was assessed with the Shapiro–Wilk test and visual inspection of histograms and Q–Q plots, with skewness and kurtosis within ±2 and ±7 judged as no serious departure [23]. Stiffness (S), tone (F), and CoAct met this criterion and were analyzed parametrically. The decrement (D) violated normality (skewness/kurtosis beyond ±2/±7; Shapiro–Wilk *p* < 0.05 in all conditions) and was therefore cross-checked with nonparametric tests alongside the parametric analysis.

Changes in D, S, F, and CoAct according to stance condition (BGS, UBS) and repetition count (1, 5, 10) were analyzed using a 2 × 3 two-way repeated-measures ANOVA. CCI was introduced as a complementary index to CoAct (Section 2.3) rather than as an independent primary outcome; because the two indices are mathematically related quantifications of the same underlying co-contraction construct, entering both into the confirmatory model would be statistically redundant, so CoAct was retained as the primary co-contraction outcome in this ANOVA and CCI was examined descriptively and in the exploratory correlation and subgroup analyses reported below. To confirm that this exclusion did not obscure a genuine effect, the same 2 × 3 repeated-measures ANOVA was additionally computed for CCI: neither the stance main effect (F(1, 26) = 0.29, *p* = 0.597, ηp^2^ = 0.011), the repetition main effect (F(2, 52) = 0.77, *p* = 0.470, ηp^2^ = 0.029), nor their interaction (F(2, 52) = 0.02, *p* = 0.977, ηp^2^ = 0.001) was significant, mirroring the null pattern observed for CoAct and confirming that both co-contraction indices remained stable across conditions. Sphericity was checked with Mauchly’s test and Greenhouse–Geisser correction applied when violated; significant main effects were followed by Bonferroni-corrected pairwise comparisons, and effect sizes were partial eta squared (ηp^2^). For D, the repetition effect was additionally verified with the Friedman test and Bonferroni-corrected Wilcoxon signed-rank post hoc tests. MG-LG differences were tested with a paired t-test, and muscle-onset rank order with the Friedman test followed by Wilcoxon comparisons. The relationship between movement time and viscoelasticity/co-contraction was analyzed with Pearson and Spearman correlations (the Spearman coefficient preferred for pairs involving D), and a fast/slow group comparison (median split on 10-rep movement time) used independent-samples t-tests with Levene’s test. The movement-time analyses were treated as exploratory. All significance levels were α = 0.05.

## 3. Results

### 3.1. Changes in Achilles Tendon Viscoelasticity

Descriptive statistics of the MyotonPRO viscoelastic variables by stance condition and repetition count are presented in Table 2. In the two-way repeated-measures ANOVA (Table 3), the repetition-count main effect was significant for the decrement (D) (F(2, 52) = 4.68, *p* = 0.014, ηp^2^ = 0.153). The main effect of stance condition (F(1, 26) = 0.46, *p* = 0.506, ηp^2^ = 0.017) and the condition × repetition interaction (F(2, 52) = 2.15, *p* = 0.127, ηp^2^ = 0.076) were not significant; that is, the pattern of change in D with repetition was similar in the two stance conditions. Notably, D rose transiently after a single BGS repetition (M = 0.982) relative to baseline (M = 0.970) before decreasing progressively with further repetitions (Table 2); this initial, non-monotonic pattern is addressed mechanistically in Section 4.1.

In the Bonferroni-corrected post hoc comparisons (Table 4), pooled across conditions, D decreased progressively as the repetition count increased (1 rep 0.973, 5 reps 0.959, 10 reps 0.937), with the difference between 1 and 10 repetitions being significant (*p* = 0.020); 1 vs. 5 (*p* = 0.626) and 5 vs. 10 (*p* = 0.260) were not significant. Because a lower D indicates higher elastic-recovery efficiency, this suggests that Achilles tendon elasticity improved as high-speed repetitions accumulated. Because D violated normality, the same effect was re-verified nonparametrically: the Friedman test was significant (χ^2^(2) = 6.75, *p* = 0.034, Kendall’s W = 0.13), and the Wilcoxon post hoc test also showed a significant 1-vs-10 difference (Bonferroni *p* = 0.013), consistent with the parametric analysis.

For stiffness (S) and tone (F), no effect of stance condition, repetition count, or interaction was significant (S: all *p* ≥ 0.536; F: all *p* ≥ 0.302) (Table 3). Achilles tendon stiffness and tone were thus stably maintained across the two stance conditions and repetition counts.

### 3.2. Changes in Ankle Agonist–Antagonist Co-Activation

The MG and LG did not differ significantly in across-condition mean activation (MG 89.73 ± 28.06%MVIC, LG 88.50 ± 29.04%MVIC; t(26) = 0.20, *p* = 0.840) (Table 5); the two heads were therefore averaged into a single gastrocnemius (agonist) variable. Descriptive statistics of sEMG activation and the co-activation index are presented in Table 6. The TA was ≈49–53%MVIC and the gastrocnemius ≈86–91%MVIC across conditions, with CoAct distributed in the ≈70–73% range; the CCI [22] was distributed in the ≈77–82% range.

In the two-way repeated-measures ANOVA (Table 7), no effect was significant for CoAct—stance condition (F(1, 26) = 0.37, *p* = 0.551, ηp^2^ = 0.014), repetition (F(2, 52) = 0.57, *p* = 0.571, ηp^2^ = 0.021), or interaction (F(2, 52) = 0.15, *p* = 0.865, ηp^2^ = 0.006). Ankle agonist–antagonist co-activation was thus maintained constantly at ≈72% regardless of stance condition and repetition count.

### 3.3. Tissue–Neural Dissociation

In sum, as high-speed repetitions accumulated, Achilles tendon elasticity (D) improved significantly, whereas ankle agonist–antagonist co-activation (CoAct) during the same task was maintained constant; that is, tissue-level viscoelastic adaptation was expressed without an accompanying neuromuscular-level change in co-activation.

### 3.4. Relationship Between Execution Speed and Achilles Tendon Viscoelasticity

Correlations between movement time and the viscoelastic variables (D, S, F) were analyzed by stance condition and repetition count (Table 8). Condition-wise movement times were, for BGS, 0.37 ± 0.04, 2.26 ± 0.28, and 4.29 ± 0.46 s at 1, 5, and 10 reps, and, for UBS, 0.38 ± 0.05, 2.39 ± 0.36, and 4.75 ± 0.62 s. No condition reached significance (all *p* > 0.05), but D showed a positive correlation tendency with movement time in all six conditions (r = 0.149–0.328), suggesting that better elasticity (lower D) was associated with faster performance, whereas S and F were unrelated (r ≈ 0). In the per-participant-mean analysis, per-cycle movement time and mean D also showed a positive but non-significant tendency (Pearson r = 0.361, *p* = 0.064; Spearman ρ = 0.151, *p* = 0.451). The confirmatory test of the movement time–elasticity relationship specified in Section 2.4 is this per-participant-mean analysis, for which both Pearson and Spearman coefficients are reported; the six condition-wise correlations in Table 8 are presented as a supplementary, descriptive breakdown using Pearson coefficients to illustrate the pattern across individual conditions and are not themselves the pre-specified confirmatory statistic.

### 3.5. Characteristics of Fast-Performing Athletes

Because the median split was performed separately on the 10-repetition movement time within each stance condition, the fast (*n* = 13) and slow (*n* = 14) groups were defined independently for BGS and UBS and did not necessarily comprise the same participants across the two conditions. Within each condition, the resulting fast and slow groups were compared with independent-samples t-tests (Table 9), and all variables reflect values measured under the 10-repetition condition. No significant between-group differences were observed in either condition (all *p* > 0.05). Although several numerical differences were descriptively noted—for example, numerically higher MG activation in the fast group (BGS *p* = 0.130, UBS *p* = 0.088) and, in BGS, numerically lower decrement (D, *p* = 0.138), higher CCI (*p* = 0.144), and higher gastrocnemius activation (*p* = 0.113)—none reached statistical significance and these values should not be interpreted as directional effects. CoAct, stiffness, and tone did not differ between groups.

### 3.6. Muscle Activation Onset Rank

The activation onset rank of the three muscles (TA, MG, LG) differed significantly in the Friedman test (χ^2^(2) = 11.73, *p* = 0.003, Kendall’s W = 0.217) (Table 10). The mean ranks were MG (1.48) < LG (2.19) < TA (2.33), indicating that the medial gastrocnemius was activated first; in the Wilcoxon post hoc tests, the MG significantly preceded both the TA (Z = −3.05, *p* = 0.002) and the LG (Z = −2.64, *p* = 0.008) (Bonferroni α = 0.017), whereas the TA and LG did not differ (*p* = 0.461). The onset-leader proportion of each muscle did not differ between stance conditions (all *p* > 0.05), so the agonist (gastrocnemius)-leading pattern was consistent regardless of condition. That is, rather than antagonist-led pre-stabilization, an agonist-driven feedforward priming pattern was observed.

### 3.7. Relationships Among Co-Contraction, Elasticity, and Execution Speed

Correlations among CCI, CoAct, elasticity (D), and movement time were analyzed by stance condition (Table 11). In BGS, CCI showed a negative correlation with movement time (Spearman ρ = −0.411, *p* = 0.033; Pearson r = −0.367, *p* = 0.060), indicating a tendency for faster performance with greater co-contraction; this was not observed in UBS (*p* > 0.05). CoAct was unrelated to execution speed in both conditions. Neither CCI nor CoAct correlated significantly with elasticity (D) in either condition (*p* > 0.05). Thus, neural-level co-contraction/co-activation and tissue-level viscoelasticity varied independently, re-confirming the tissue–neural dissociation of Section 3.3 at the correlation level. Given that 24 correlation coefficients were examined across variable pairs, conditions, and estimation methods, and only one (the BGS CCI–movement-time correlation) reached significance, this single result should be interpreted with caution as preliminary rather than a confirmed association, pending correction for multiple comparisons in future work.

## 4. Discussion

Regarding the hypotheses stated in the Introduction: the hypothesis that Achilles tendon elasticity would improve (lower decrement) with accumulating high-speed repetitions was supported, as D decreased significantly with repetition (Section 3.1). The exploratory question of whether this tissue-level adaptation would be accompanied by a change in co-contraction was answered in the negative: co-contraction (CoAct, CCI) remained constant across conditions, indicating a tissue–neural dissociation rather than coupled adaptation (Section 3.2 and Section 3.3). The a priori hypothesis that the less stable, single-limb-loading UBS condition would elicit greater co-contraction and a larger viscoelastic change than BGS was not supported: neither the stance main effect for D (F(1, 26) = 0.46, *p* = 0.506) nor co-contraction differed meaningfully between stances (Section 3.1 and Section 3.3). Finally, the hypothesis that shorter movement time would be associated with higher elasticity was likewise not confirmed: although a positive tendency was observed, the pooled correlation did not reach significance (Pearson r = 0.361, *p* = 0.064; Spearman ρ = 0.151, *p* = 0.451; Section 3.4).

### 4.1. Acute Enhancement of Achilles Tendon Elasticity with High-Speed Repetition

The primary finding is that the Achilles tendon decrement (D) decreased significantly as high-speed repetitions accumulated (1 rep 0.973 → 10 reps 0.937, *p* = 0.020; Friedman χ^2^(2) = 6.75, *p* = 0.034). Because D reflects the dissipation of mechanical energy during recovery after deformation, its decrease means that the energy lost per unit deformation decreased and elastic-recovery efficiency increased. Within the same stance condition, the Achilles tendon was thus acutely reorganized toward more efficient storage and release of elastic energy as repetitions accumulated.

This acute change is better interpreted as tissue preconditioning and a change in viscoelastic behavior due to repetitive loading than as chronic training adaptation. Repetitive stretch–shortening loading induces water redistribution within the MTU, a decrease in the viscous component, a rise in tissue temperature, and collagen-fiber alignment and crimp reduction, thereby lowering hysteresis loss [24,25,26]; because damping is sensitive to such viscous/fluid components, the finding that D changed first is mechanically consistent. This accords with the view that elastic energy in explosive movements is stored and released primarily in the tendon [2,4,27] and with reports that tendon mechanical properties are reorganized variably with loading history and intensity [5]. Consistent with this preconditioning-based account, D rose transiently after a single BGS repetition (M = 0.982) relative to baseline (M = 0.970) before decreasing progressively with further repetitions (Table 2); such an initial, non-monotonic response is characteristic of the first cycle in cyclic-preconditioning paradigms, in which collagen-fiber recruitment and crimp straightening are not yet complete, and is expected to resolve as loading cycles accumulate [24,25].

Notably, this change in D appeared similarly in the two stance conditions (both the condition main effect and the interaction were non-significant). Although the anterior dominant-foot load proportion was differentiated by the rear support-surface height, elastic enhancement was driven by repetition itself rather than by load distribution, suggesting that acute elastic adaptation may be a general tissue response to repetitive high-speed loading rather than a stance-specific phenomenon.

### 4.2. Stability of Stiffness and Tone

In contrast, stiffness (S) and tone (F) showed no significant change for any effect. Short-term repetitive loading thus did not alter the structural stiffness of the Achilles tendon or its intrinsic resting tension. The acute adaptation observed here was confined to the viscous/damping dimension rather than to structural stiffness, and the fact that, among the MyotonPRO’s multidimensional indices, D selectively captured this change supports the study’s distinctiveness in providing information difficult to capture directly with ultrasound morphological variables.

### 4.3. Co-Contraction Maintained Regardless of Stance and Repetition

Ankle co-activation (CoAct) was maintained constantly at ≈72% regardless of stance and repetition, and CCI was likewise stable in the 77–82% range, indicating that the nervous system maintained a constant level of joint stiffness throughout the task [28]. Given that the task simultaneously required single-foot balance, landing accuracy, and center-of-mass control, the high co-contraction of ≈72% can be regarded as a stability-first strategy [18,28,29]. That this level did not change with repetition indicates a preemptively set co-contraction ‘baseline’ established at task onset and maintained throughout the repetitions [28,30] rather than trial-by-trial fine adjustment.

### 4.4. Muscle Onset Order and Feedforward Priming

The onset-rank analysis showed that the medial gastrocnemius was consistently activated first (MG < LG < TA), significantly preceding both the TA and the LG, and this agonist-leading order did not differ between stance conditions. Rather than antagonist-led pre-stabilization, this indicates an agonist-driven feedforward priming pattern, in which the gastrocnemius is pre-activated to prepare the propulsive stretch–shortening action before the antagonist contributes to joint stabilization [28,30]. This agonist-first sequence is compatible with the constant co-contraction baseline above: the nervous system appears to establish a stable antagonistic background while prioritizing early agonist drive for performance.

### 4.5. Tissue–Neural Dissociation: Core Interpretation

The most important finding is the ‘tissue–neural dissociation’: tissue-level elasticity (D) improved significantly with repetition, whereas neuromuscular co-activation was maintained constant. At the correlation level, neither CCI nor CoAct related significantly to D in either condition (*p* > 0.05), so the dissociation held in both change scores and covariance structure. This suggests that the acute elastic adaptation of the Achilles tendon is not mediated by changes in neuromuscular co-contraction but is expressed independently as a peripheral, tissue-level mechanical phenomenon: co-contraction provides a constant neural base ensuring joint stability, upon which tendon viscoelastic efficiency is separately improved with repetition—a two-layered structure. This partially reconsiders, in an acute context, the assumption that tendon mechanics and neuromuscular control are tightly coupled [16,17,31,32,33], and could be observed separately only because the present design applied MyotonPRO and surface electromyography simultaneously.

### 4.6. Execution Speed, Co-Contraction, and Elasticity (Exploratory)

In BGS, CCI correlated negatively with movement time (Spearman ρ = −0.411, *p* = 0.033), indicating faster performance with greater co-contraction; this was absent in UBS. As a non-significant trend, the fast group also showed lower D, higher CCI, and higher gastrocnemius activation in BGS. The relatively stable BGS may have allowed appropriate co-contraction to stiffen the joint and assist the use of elastic energy in the stretch–shortening cycle, enabling faster performance [28,29]; in UBS, the greater balance demand may have mobilized co-contraction mainly for stability, weakening its link to speed. Except for the BGS CCI–time correlation, these relationships were non-significant and, given the sample size (*n* = 27) and manual-marking resolution (≈8.3 ms), should be interpreted cautiously.

### 4.7. Limitations

First, as an acute single-session design, the results cannot be generalized to long-term training adaptation. Second, the stance-condition order was fixed (BGS first) for safety, so order/fatigue effects could not be fully excluded (mitigated by 3-min rest). In addition, within each stance the 1–5–10 repetition order was likewise fixed (not randomized) and identical for all participants, so cumulative fatigue or potentiation across repetitions cannot be fully disentangled from the repetition-count effect itself. Third, the sample was limited to male collegiate American football players, spanning a wide BMI range (21.5–39.4 kg/m^2^, extending into the obese classification); this single-sex, single-sport composition together with this BMI heterogeneity limits generalizability to women, other sports, and non-athletic populations. Fourth, the MyotonPRO measures only local viscoelasticity at a single site and does not directly measure in vivo tendon strain/tension, and concurrent ultrasound was not performed. Fifth, the surface-EMG-based co-contraction index is an amplitude-based estimate, not a direct measure of joint stiffness [34]. Sixth, although D was cross-checked nonparametrically, distributional limitations remain. Seventh, although the final sample (*n* = 27) exceeded the a priori power requirement for the primary repeated-measures analyses, the overall sample size remains modest. American football has a relatively small participant population in Korea, with only a limited number of collegiate teams nationwide, and eligible players had to be recruited without interfering with each team’s in-season training and competition schedule; these practical constraints made recruitment across multiple teams and institutions difficult and restricted the feasible sample to a single institution. Consequently, statistical power to detect smaller inter-individual effects remains limited, and generalizability is constrained; the findings should therefore be regarded as preliminary pending replication in larger, multi-institution samples as the athlete pool allows. Eighth, participants’ BMI varied widely (21.5–39.4 kg/m^2^), most likely reflecting the natural body-composition heterogeneity across American-football playing positions rather than a data anomaly; however, because percentage body fat was not measured, whether the higher-BMI participants carried greater lean versus fat mass could not be verified, and BMI could not be examined as a covariate. A BMI-matched non-sportive control group was not included, as the within-subject repeated-measures design was intended to isolate the acute within-tendon effect of loading condition using each participant as their own baseline, given evidence that chronic mechanical loading history is associated with substantial adaptive changes in tendon stiffness and material properties (meta-analytic effect size ≈0.70 for loading-induced stiffness change) [35], such that comparing highly trained athletes with non-sportive individuals would introduce additional baseline (training-history) heterogeneity not aligned with this acute, within-subject aim; such a control group, together with direct body-composition assessment, should be considered in future studies. Ninth, no validated minimal detectable change value for the decrement (D) exists in the literature for this measurement site, so the magnitude of the observed group-level D change cannot be benchmarked against a measurement-error threshold, and this small effect should be interpreted with this caveat in mind. Finally, the execution-speed analyses are exploratory and limited in power.

### 4.8. Practical Applications

High-speed repetitive movement acutely raised Achilles tendon elastic efficiency without perturbing neuromuscular co-contraction. In warm-up/priming design preceding explosive performance, repetitive high-speed loading may therefore serve to make tendon elasticity more efficient while preserving stability (constant co-contraction). Because stance/load design (BGS ⟷ UBS) differentiates the anterior single-foot load proportion without changing neural co-contraction, it offers a practical basis for designing load modulation and neural control separately.

## 5. Conclusions

In 27 male collegiate American football players, MyotonPRO and surface electromyography were applied simultaneously during high-speed repetitive movements combining two stance conditions and three repetition counts, analyzing Achilles tendon viscoelasticity and ankle agonist–antagonist co-contraction in an integrated manner. As repetitions accumulated, the Achilles tendon decrement (D) decreased significantly and elastic-recovery efficiency improved acutely, whereas stiffness (S) and tone (F) remained stable; ankle co-activation (CoAct) was maintained constantly at ≈72% regardless of stance and repetition; the medial gastrocnemius was consistently activated first, indicating agonist-driven feedforward priming; and a tissue–neural dissociation—tissue-level elastic adaptation without neuromuscular-level change in co-contraction—was confirmed in both change scores and correlation structure. These findings show that the acute elastic enhancement of the Achilles tendon is expressed independently as a tissue-level mechanical phenomenon rather than being mediated by neuromuscular co-contraction, and demonstrate that combined MyotonPRO–sEMG analysis provides functional information beyond existing imaging techniques. The study can serve as foundational data for stance design and load-modulation strategies and for the development of Achilles tendon injury-prevention programs. Future research should use longitudinal designs, extend to women and other populations, and directly verify the tissue–neural dissociation mechanism with concurrent ultrasound.

## Figures and Tables

**Table 1:** Characteristics of study participants (*n* = 27).

Variable	Mean ± SD	Range
Age (years)	23.19 ± 2.63	19–28
Height (cm)	177.26 ± 6.35	166.5–194.1
Weight (kg)	86.10 ± 15.16	65.5–132.9
Body mass index (kg/m^2^)	27.35 ± 4.08	21.5–39.4
Lower-leg length (cm)	42.11 ± 2.19	39.0–48.0

Note. Values are means ± SD. All participants were male collegiate American-football players. Dominant foot: right = 22, left = 5.

**Table 2:** Descriptive statistics of Achilles tendon viscoelastic variables by stance condition and repetition.

Condition	D (Decrement)	S (N/m)	F (Hz)
Baseline	0.970 ± 0.179	1123.07 ± 210.95	33.12 ± 3.74
BGS—1	0.982 ± 0.182	1170.30 ± 151.53	34.59 ± 3.52
BGS—5	0.952 ± 0.149	1215.11 ± 255.40	34.90 ± 5.24
BGS—10	0.921 ± 0.137	1191.22 ± 223.75	33.87 ± 4.11
UBS—1	0.964 ± 0.150	1168.85 ± 153.81	33.76 ± 2.87
UBS—5	0.965 ± 0.181	1191.15 ± 195.90	34.36 ± 4.16
UBS—10	0.952 ± 0.126	1207.74 ± 187.50	34.49 ± 3.74

Note. Values are mean ± SD. D = decrement (lower = greater elasticity); S = stiffness; F = tone (frequency). BGS = bilateral ground stance; UBS = unilateral box stance. Measured on the dominant limb in the prone position immediately after each condition.

**Table 3:** Two-way (condition × repetition) repeated-measures ANOVA of Achilles tendon viscoelastic variables.

Variable	Effect	F	df	*p*	ηp^2^
D (decrement)	Condition	0.46	1, 26	0.506	0.017
	Repetition	4.68	2, 52	0.014 *	0.153
	Condition × Repetition	2.15	2, 52	0.127	0.076
S (N/m)	Condition	0.01	1, 26	0.911	0.000
	Repetition	0.63	2, 52	0.536	0.024
	Condition × Repetition	0.30	2, 52	0.742	0.011
F (Hz)	Condition	0.28	1, 26	0.603	0.011
	Repetition	0.34	2, 52	0.712	0.013
	Condition × Repetition †	1.23	2, 52	0.302	0.045

Note. ηp^2^ = partial eta squared. * *p* < 0.05. † Sphericity violated (Mauchly, *p* < 0.05); Greenhouse–Geisser-corrected *p* reported. D = decrement; S = stiffness; F = tone.

**Table 4:** Bonferroni-corrected pairwise comparisons for the repetition main effect on decrement (D), collapsed across conditions.

Comparison (Reps)	Mean Difference	*p* (Bonferroni)
1 vs. 5	0.014	0.626
1 vs. 10	0.036	0.020 *
5 vs. 10	0.022	0.260

Note. Mean difference = earlier − later repetition (positive = decrease in D, i.e., increased elasticity). Pooled D: 1 rep = 0.973, 5 reps = 0.959, 10 reps = 0.937. * *p* < 0.05.

**Table 5:** Comparison of medial versus lateral gastrocnemius activation (paired-samples *t*-test).

Muscle Head	Mean ± SD (%MVIC)	t (df)	*p*
Medial gastrocnemius (MG)	89.73 ± 28.06	0.20 (26)	0.840
Lateral gastrocnemius (LG)	88.50 ± 29.04		

Note. Values are each participant’s mean activation (%MVIC) averaged across all six conditions. The two heads did not differ significantly, supporting their combination into a single gastrocnemius (agonist) variable.

**Table 6:** Descriptive statistics of surface-EMG activation and co-activation by stance condition and repetition (*n* = 27).

Condition	TA	MG	LG	Gastrocnemius	CoAct	CCI
BGS—1	52.52 ± 14.54	88.10 ± 23.83	85.53 ± 29.63	86.81 ± 23.50	72.36 ± 10.30	79.99 ± 23.66
BGS—5	50.01 ± 13.47	84.85 ± 23.30	87.84 ± 30.99	86.35 ± 23.61	71.55 ± 12.01	76.82 ± 23.51
BGS—10	50.92 ± 12.21	92.15 ± 48.76	87.26 ± 29.49	89.70 ± 31.08	72.50 ± 13.76	79.85 ± 24.94
UBS—1	51.01 ± 12.80	91.31 ± 26.14	91.45 ± 34.97	91.38 ± 26.56	72.36 ± 12.03	81.48 ± 24.77
UBS—5	48.80 ± 14.70	89.71 ± 28.75	91.81 ± 31.46	90.76 ± 26.97	70.31 ± 13.75	77.61 ± 29.90
UBS—10	52.74 ± 21.14	92.25 ± 38.10	87.10 ± 32.34	89.67 ± 27.37	72.11 ± 12.16	81.96 ± 33.63

Note. Values are mean ± SD. TA, MG, LG, and Gastrocnemius in %MVIC; CoAct and CCI in %. Gastrocnemius = mean of MG and LG (agonist). CoAct = co-activation index [16]; CCI = co-contraction index [22]. BGS = bilateral ground stance; UBS = unilateral box stance.

**Table 7:** Two-way (condition × repetition) repeated-measures ANOVA of the co-activation index (CoAct).

Effect	F	df	*p*	ηp^2^
Condition	0.37	1, 26	0.551	0.014
Repetition †	0.57	2, 52	0.571	0.021
Condition × Repetition †	0.15	2, 52	0.865	0.006

Note. ηp^2^ = partial eta squared. No effect reached significance. † Sphericity violated (Mauchly, *p* < 0.05); Greenhouse–Geisser-corrected *p* reported.

**Table 8:** Correlations between movement time and Achilles tendon viscoelasticity (D, S, F) by condition.

Condition	Time–D (r; ρ)	Time–S	Time–F
BGS—1	0.149 (0.458) ρ = −0.032 (0.876)	−0.044 (0.827)	−0.056 (0.780)
BGS—5	0.243 (0.222) ρ = 0.302 (0.126)	0.001 (0.995)	−0.049 (0.808)
BGS—10	0.275 (0.165) ρ = 0.186 (0.354)	−0.184 (0.357)	−0.179 (0.371)
UBS—1	0.328 (0.095) ρ = 0.098 (0.626)	−0.014 (0.946)	−0.140 (0.486)
UBS—5	0.325 (0.098) ρ = 0.057 (0.776)	−0.059 (0.769)	−0.055 (0.785)
UBS—10	0.313 (0.112) ρ = 0.135 (0.501)	−0.002 (0.993)	−0.022 (0.913)

Note. Values are Pearson r (two-tailed *p*). *n* = 27. None significant. A positive r indicates that higher D (lower elasticity) is associated with longer movement time. Spearman ρ (second line of each Time–D cell) is additionally reported for consistency with the non-parametric analysis plan for D (Section 2.4); all six values are likewise non-significant and consistent in direction with the Pearson coefficients. Pooled subject-mean analysis (per-cycle time × mean D): r = 0.361, *p* = 0.064; Spearman ρ = 0.151, *p* = 0.451. BGS = bilateral ground stance; UBS = unilateral box stance.

**Table 9:** Comparison of fast and slow groups under the 10-repetition condition, by stance (median split on 10-rep movement time, performed separately within each stance).

Variable	Fast (M ± SD)	Slow (M ± SD)	t	*p*
*Bilateral ground stance (BGS)*				
D	0.88 ± 0.08	0.96 ± 0.17	−1.53	0.138
S	1203.8 ± 169.9	1179.6 ± 270.5	0.28	0.785
F	33.8 ± 3.6	34.0 ± 4.7	−0.14	0.890
CCI	87.2 ± 27.2	73.0 ± 21.4	1.51	0.144
CoAct	72.7 ± 16.6	72.3 ± 11.2	0.06	0.951
Gastrocnemius	99.6 ± 35.4	80.5 ± 24.2	1.64	0.113
TA	53.9 ± 11.2	48.1 ± 12.9	1.25	0.224
MG	107.0 ± 63.3	78.4 ± 25.0	1.57	0.130
LG	92.2 ± 27.7	82.7 ± 31.4	0.83	0.414
*Unilateral box stance (UBS)*				
D	0.94 ± 0.10	0.96 ± 0.15	−0.46	0.647
S	1240.9 ± 246.7	1177.0 ± 109.6	0.88	0.387
F	34.9 ± 4.9	34.1 ± 2.4	0.61	0.551
CCI	85.4 ± 26.1	78.8 ± 40.1	0.50	0.621
CoAct	72.0 ± 13.3	72.2 ± 11.5	−0.06	0.955
Gastrocnemius	96.0 ± 29.0	83.8 ± 25.4	1.17	0.251
TA	55.0 ± 14.6	50.6 ± 26.2	0.54	0.595
MG	105.2 ± 44.9	80.2 ± 26.8	1.77	0.088
LG	86.9 ± 34.1	87.3 ± 31.9	−0.03	0.974

Note. Fast group *n* = 13, slow group *n* = 14, defined by a within-condition median split; group membership may differ between BGS and UBS. All values are from the 10-repetition condition. No comparison reached significance (all *p* > 0.05). D = decrement; S = stiffness (N/m); F = tone (Hz); CCI = co-contraction index [22]; CoAct = co-activation index; TA = tibialis anterior, MG = medial gastrocnemius, LG = lateral gastrocnemius, Gastrocnemius = MG–LG mean (all %MVIC). Homogeneity of variance was assessed with Levene’s test for each comparison and was satisfied in all cases (all *p* > 0.05); equal-variances independent-samples *t*-tests are therefore reported.

**Table 10:** Friedman test and pairwise comparisons of muscle activation onset rank (*n* = 27).

Comparison	Statistic	*p*
Friedman test (3 muscles)	χ^2^(2) = 11.73	0.003
Kendall’s W	0.217	—
MG vs. TA	Z = −3.05	0.002
MG vs. LG	Z = −2.64	0.008
LG vs. TA	Z = −0.74	0.461

Note. Mean ranks (lower = earlier onset): MG 1.48, LG 2.19, TA 2.33. Pairwise comparisons by Wilcoxon signed-rank test (Bonferroni-corrected α = 0.017). The onset-leader proportion did not differ between conditions (BGS vs. UBS): MG Z = −1.29 (*p* = 0.197), TA *p* = 0.768, LG *p* = 0.432.

**Table 11:** Correlations among co-contraction (CCI), co-activation (CoAct), elasticity (D), and movement time by condition.

Variable Pair	BGS Pearson	BGS Spearman	UBS Pearson	UBS Spearman
Time–D	0.275 (0.165)	0.186 (0.354)	0.313 (0.112)	0.135 (0.501)
Time–CCI	−0.367 (0.060)	−0.411 (0.033) *	0.027 (0.894)	−0.083 (0.682)
Time–CoAct	−0.009 (0.966)	0.054 (0.789)	0.053 (0.794)	0.006 (0.977)
CCI–D	0.159 (0.429)	−0.055 (0.786)	0.209 (0.296)	0.192 (0.338)
CoAct–D	0.161 (0.423)	0.120 (0.551)	0.146 (0.467)	0.217 (0.276)
CCI–CoAct	0.655 (0.000) **	0.526 (0.005) **	0.645 (0.000) **	0.624 (0.001) **

Note. Values are correlation coefficients (two-tailed *p*). * *p* < 0.05, ** *p* < 0.01. *n* = 27. Spearman coefficients are preferred for pairs involving D because of its non-normal distribution. BGS = bilateral ground stance; UBS = unilateral box stance.

## Data Availability

The data presented in this study are available on request from the corresponding author.
